# A randomized controlled study of remimazolam in preschool children undergoing adenotonsillectomy

**DOI:** 10.3389/fphar.2025.1678650

**Published:** 2025-10-13

**Authors:** Yong Wu, Fenjun Wang, Kai Zhu, Li Ling, Wangping Zhang

**Affiliations:** ^1^ Department of ENT, Jiaxing Hospital of Traditional Chinese Medicine, Jiaxing University, Jiaxing, China; ^2^ Department of Anesthesiology, The Affiliated Hospital of Military Medical University, Xian, China; ^3^ Department of Internal Medicine, Women and Children’s Hospital of Jiaxing University, Jiaxing, China; ^4^ Department of Anesthesiology, The First Hospital Affiliated to Gannan Medical University, Gannan, China; ^5^ Department of Anesthesiology, Women and Children’s Hospital of Jiaxing University, Jiaxing, China

**Keywords:** remimazolam, propofol, children, general anesthesia, emergence agitation

## Abstract

**Background:**

At present, remimazolam is widely used for general anesthesia. However, the literature on the usability of remimazolam is limited in preschool children. This study aimed to explore effects of remimazolam on emergence agitation and adverse events in preschool children undergoing adenotonsillectomy.

**Methods:**

A total of 100 children undergoing adenotonsillectomy, randomly divided into the Remimazolam group and the Propofol group, with 50 cases in each group. The Remimazolam group received remimazolam for the induction and maintenance of general anesthesia, and the Propofol group received propofol for the induction and maintenance of general anesthesia. The onset time, extubation time and awakening time were recorded. Systolic blood pressure (SBP), diastolic blood pressure (DBP) and heart rate (HR) were measured before anesthesia (T0), immediately before intubation (T1), 3 min after intubation (T2), 15 min after the start of the surgery (T4) and at the end of the surgery (T5). The drug-related complications including hypotension, bradycardia, injection site pain, respiratory depression, emergence agitation, nausea and vomiting were recorded.

**Results:**

The onset time was significantly longer in the Remimazolam group (64.3 ± 8.1 vs. 38.3 ± 4.5 s, *P* < 0.001), while the extubation time and awakening time were shorter in the Remimazolam group than the Propofol group (12.9 ± 2.2 vs. 14.5 ± 3.2 min, *P* = 0.005; 19.9 ± 4.7 vs. 21.8 ± 4.5 min, *P* = 0.039, respectively). The incidence of emergence agitation, hypotension and injection site pain was lower in the Remimazolam group than the Propofol group (12% vs. 30%, *P* = 0.027; 26% vs. 48%, *P* = 0.023; 4% vs. 48%, *P* < 0.001, respectively).

**Conclusion:**

This study demonstrated that remimazolam not only shortened extubation and awakening times but also reduced the incidence of emergence agitation, hypotension, and injection site pain in preschool children undergoing adenotonsillectomy compared with propofol.

**Clinical Trial Registration:**

https://www.chitr.org.com, identifier: ChiCTR240085456.

## 1 Introduction

Adenotonsillectomy is a quick surgical procedure performed on children under general anesthesia (Huh et al., 2017). Anesthetics, analgesics, and muscle relaxants may not be fully metabolized by the time the surgery is completed. Early extubation can lead to complications such as airway-related complications (Vitale et al., 2022; Serafim et al., 2022). Intravenous anesthetics include midazolam, propofol, etomidate, ketamine, and so on. At present, etomidate and ketamine are less commonly used in clinical practice due to their associated adverse effects. Compared with remimazolam, midazolam has a longer duration of action and recovery time (Kim et al., 2023). Remimazolam, a novel ultra-short-acting benzodiazepine, is characterized by its rapid onset and short duration of action. The action time and recovery time of midazolam are slower than those of remifentanil Its metabolism is dependent on non-specific cholinesterase in plasma and does not rely on liver or kidney function (Gao et al., 2023; Wiltshire et al., 2012). Furthermore, it can be quickly reversed by flumazenil (Yamamoto et al., 2021). Currently, the literature regarding the usability of remimazolam in the pediatric population is limited. Therefore, this study explored the usability and adverse events of remimazolam in preschool children undergoing adenotonsillectomy. We hypothesized that remimazolam was superior to propofol regarding extubation and awakening times and associated complications.

## 2 Materials and methods

This study followed the Declaration of Helsinki and was approved by the Hospital Ethical Committee (approval number: 2024-108). Written informed consent was obtained from the children’s parents and this trial was registered at the Chinese Clinical Trial Registry (Registration number: ChiCTR2400085436). A total of 100 children undergoing tonsillectomy with American Society of Anesthesiologists (ASA) stage II, weight <50 kg, and age 3–6 years were included this study. Exclusion criteria: children with allergy to study drugs, serious liver and kidney dysfunction, neurological diseases, and congenital heart disease.

### 2.1 Randomization

Randomization was carried out by opening an opaque, sealed envelope containing a sequential number. The allocation sequence was generated using random permuted block randomization. The investigators, surgeons, and nurses were blinded to the study. Study drugs were prepared by an anesthesiologist who was not involved in this study. Children were randomly divided into a Remimazolam group and a Propofol group using a random number table, with 50 cases in each group.

### 2.2 Anesthesia method

All children did not receive premedications. Intravenous access was established before entering the operating room. Arriving at the operating room, routine monitoring including electrocardiogram (ECG), blood pressure (BP), pulse oxygen saturation (SpO_2_), and heart rate (HR) was conducted. Anesthesia was induced in the Remimazolam group with remimazolam (Jiangsu Hengrui Medical Co., Ltd., China) 0.3 mg/kg over 60 s, sufentanil 0.3 μg/kg and cisatracurium 0.1 mg/kg. In the Propofol group, anesthesia was induced using propofol 3 mg/kg over 60 s, sufentanil 0.3 μg/kg and cisatracurium 0.1 mg/kg. Anesthesia was maintained using remimazolam 1 mg.kg-1.h-1 (in Remimazolam group) or propofol 10 mg kg^-1^.h^-1^ (in Propofol group) with remifentanil at a rate of 0.1–0.3 μg kg^-1^·min^-1^ to maintain systolic blood pressure within a 20% range of baseline and keep the bispectral index value between 45 and 55 (BIS monitor Model A2000; Aspect, USA). Endotracheal intubation was performed using direct laryngoscopy. Following intubation, the lungs were mechanically ventilated using pressure-controlled ventilation mode. The parameters were set as follows: driving pressure of 15–18 cmH_2_O, targeted tidal volume of 8 mL/kg, respiratory rate of 16–20 breaths/min, oxygen concentration of 0.6, and oxygen flow rate of 2 L/min. When the systolic blood pressure dropped by more than 20% from the baseline, ephedrine 0.2 mg/kg was administered *via* intravenous injection. Atropine 0.01 mg/kg was given if HR was less than 60 beats/min. At the end of the surgery, ondansetron 0.08 mg/kg was administered to prevent postoperative nausea and vomiting, then the children were transferred to the post-anesthesia care unit (PACU). Muscle block was antagonized with neostigmine 0.04 mg kg^−1^ and atropine 0.02 mg kg^−1^. The criteria for extubation were as follows: spontaneous tidal volumes greater than 6 mL/kg, a respiratory rate exceeding 10 breaths/min, SpO_2_ above 96%, and end-tidal CO_2_ pressure (P_ET_CO_2_) below 50 mmHg while receiving oxygen. Children were discharged from the PACU when their Aldrete scores were greater than 9 (Zhu et al., 2021a).

### 2.3 Measurements

Preoperative anxiety scales were assessed using the Yale preoperative anxiety scale (YPAS) (Kain et al., 1995). The onset time, extubation time and awakening time were recorded. Systolic blood pressure (SBP), diastolic blood pressure (DBP) and heart rate (HR) were measured before anesthesia (T0), immediately before intubation (T1), 3 min after intubation (T2), 15 min after the start of surgery (T3), and at the end of surgery (T4). Drug-related complications, including hypotension, bradycardia, injection site pain, respiratory depression, emergence agitation, nausea and vomiting, were recorded. Emergence agitation was assessed using the Pediatric Anesthesia Emergence Delirium (PAED) scale. The PAED scale evaluates emergence agitation using five items: 1) Eye contact with the caregiver, 2) Purposeful actions, 3) Awareness of surroundings, 4) Restlessness, and 5) Inability to calm down. It employs a five-point Likert scale (0 = Extremely; 1 = Very much; 2 = Quite a bit; 3 = Just a little; 4 = Not at all) for quantifying emergence agitation, with dimensions four and five utilizing a reverse scoring system. Emergence agitation was defined as a PAED score of ≥12 (He et al., 2023).

The sedative levels were assessed using the Ramsay Sedation Score (RSS) (1, patients are anxious, agitated, or restless; 2, patients are cooperative, oriented, alert, and tranquil; 3, patients respond to commands; 4, asleep, but with rapid response to stimulus; 5, sleeping, with sluggish response to stimulus; 6, asleep, with no response) (Zhang and Li, 2018). An RSS score of >4 was considered as excessive sedation.

Onset time was defined as the time from the administration of sedatives/anesthetics to the loss of the eyelash reflex. The extubation time was the time from the end of the surgery to the eye-opening. Hypotension was defined as a systolic blood pressure that fell more than 20% below the baseline value. Bradycardia was defined as HR of less than 60 beats/min, and respiratory depression was defined as SpO_2_ < 94% whilst receiving supplemental oxygen, and a breathing rate <10 breaths/min.

### 2.4 Sample size calculation

The primary outcome of this study was the incidence of emergence agitation. Secondary outcomes were the extubation and awakening times. A pilot study with 10 patients in each group indicated that the incidence of emergence agitation was 10% and 35%, respectively. We calculated that a sample size of 44 patients in each group would have detect a difference in the incidence of emergence agitation between the two groups with an α of 0.05 and a power of 0.8. The sample size in each group was increased to 50 to account for potential dropouts.

### 2.5 Statistical analysis

Statistical analysis was performed using SPSS 22.0 software. Measurement data variables with normally distribution are expressed as mean ± standard deviation (SD) and analyzed with *t*-test or analysis of variance, Numerical variables with nonnormally distribution were expressed as median [range] and were analyzed by Mann-Whitney U test. While count variables were analyzed using the chi-square test or Fisher’s exact test. A p-value less than 0.05 was defined as statistically significant.

## 3 Results

**FIGURE 1:**
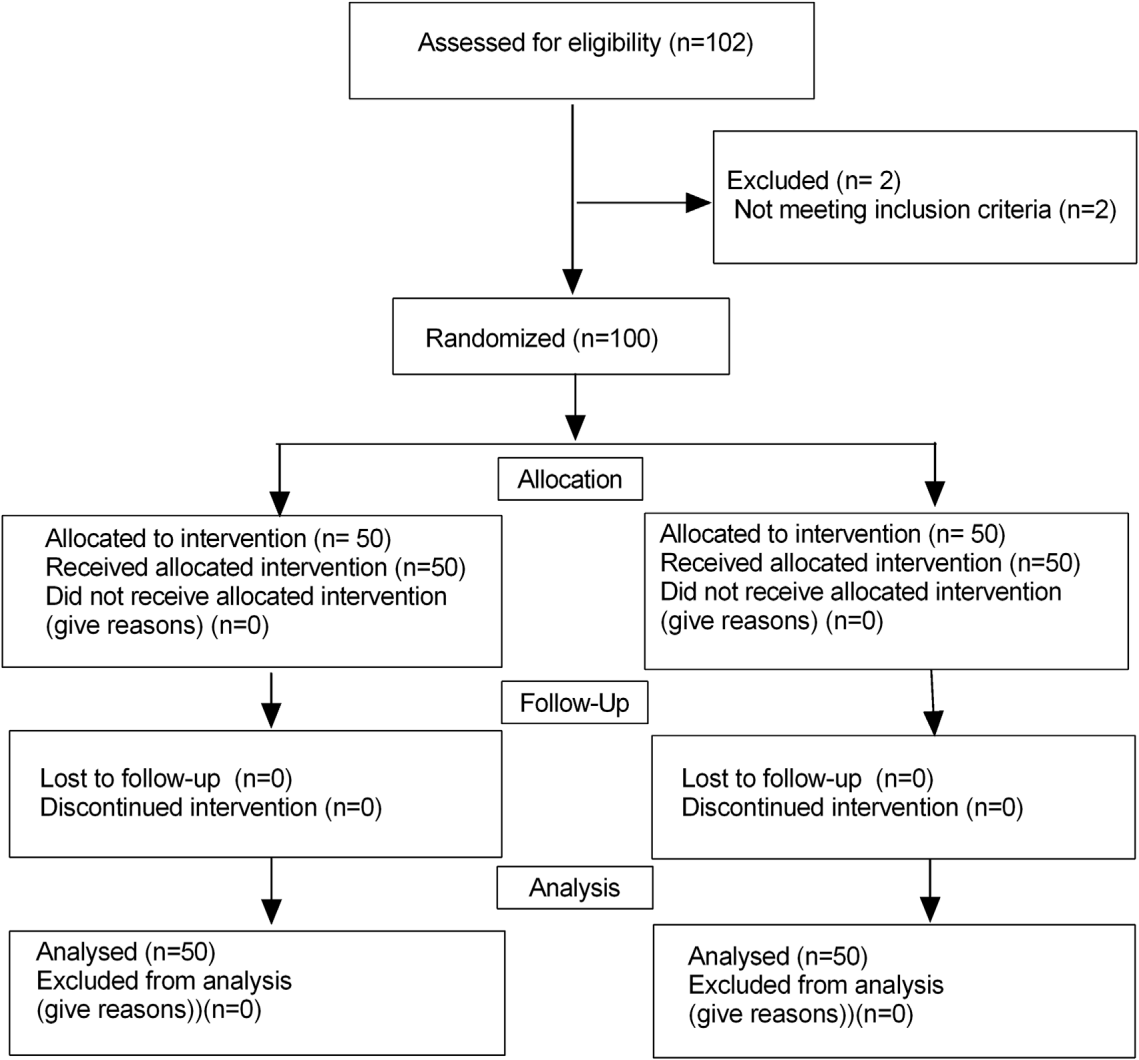
Flow diagram of study.

Initially, a total of 102 children were enrolled in this trial, and finally, 100 children finished this study (Figure 1). There were no significant differences in terms of the age, sex, weight, YPAS scores, duration of anesthesia and duration of operation, PACU time and amount of remifentanil between the two groups (*P* > 0.05) (Table 1). The onset time was significantly longer in the Remimazolam group (64.3 ± 8.1 vs38.3 ± 4.5 s, *P* < 0.001). While the extubation time and awakening time were shorter in the Remimazolam group (12.9 ± 2.2 vs14.5 ± 3.2 min, *P* = 0.005; 19.9 ± 4.7 vs. 21.8 ± 4.5 min, *P* = 0.039, respectively). PAED scores were lower in the Remimazolam group compared to the Propofol group, there were significant differences in PAED scores (*P* = 0.011).

**TABLE 1:** Characteristics of the children.

Index	Remimazolam group (*n* = 50)	Propofol group (*n* = 50)	*P*-value
Age (year)	4.9 ± 1.1	5.0 ± 1.1	0.711
Sex (Male/Female, n)	28/22	26/24	0.688
Weight (kg) YPAS score Duration of anesthesia (min) Duration of surgery (min) Extubation time (min) Awakening time (min) PACU time (min) Onset time (s) Amount of remifentanil (ug) PAED score	19.4 ± 3.7 54.6 ± 7.1 42.2 ± 7.0 31.2 ± 6.7 12.9 ± 2.2 19.9 ± 4.7 34.3 ± 4.8 64.3 ± 8.1 35.6 ± 6.5 6 [4-13]	20.3 ± 4.2 56.5 ± 7.9 42.0 ± 4.6 29.8 ± 4.7 14.5 ± 3.2 21.8 ± 4.5 34.5 ± 3.7 38.3 ± 4.5 36.8 ± 5.8 8 [5-14]	0.268 0.210 0.880 0.248 0.005* 0.039* 0.834 <0.001* 0.335 0.011*

Data are presented as mean ± SD, or numbers/[rang]. *P < 0.05. PAED: Pediatric Anesthesia Emergence Delirium. YPAS: Yale Preoperative Anxiety Scale. Duration of anesthesia was defined as the time from the administration of anesthetics to discontinuation of anesthetics. Duration of surgery was defined as the time from the start of surgery to the end of surgery. Onset time was defined as the time from the administration of sedatives/anesthetics to the loss of the eyelash reflex. The extubation time was the time from the end of the surgery to the eye-opening.

### 3.1 Hemodynamics

**FIGURE 2:**
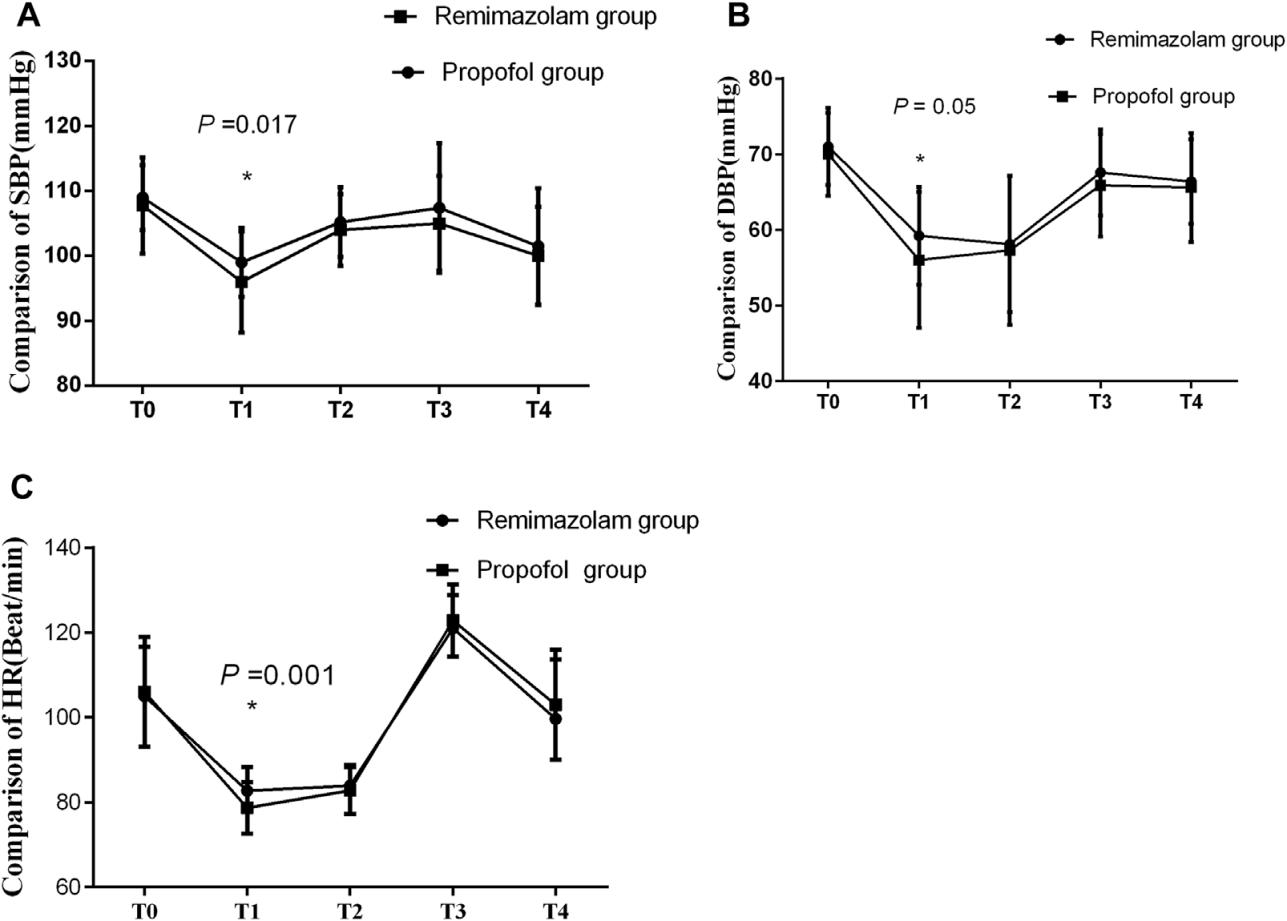
**(A–C)** Comparison of hemodynamics between the two groups. There were no significant differences in hemodynamics between the two groups. SBP: systolic blood pressure, DBP: diastolic blood pressure, HR: heart rate. T0: before anesthesia, T1: immediately before intubation, T2: 3 min after intubation, T3: 15 min after the start of the surgery and T4: at the end of the surgery.

Comparison of hemodynamics are showed in Figure 2. The SBP, DBP, and HR after the administration of sedatives/anesthetics showed a significant decrease, compared with those before administration in both groups. At T2, there were significant differences in SBP, DBP, and HR between the two groups, all *P* < 0.05, while there were no significant differences in SBP, DBP, and HR at other time-points.

### 3.2 Adverse events

Adverse events of children are showed in Table 2. The incidence of emergence agitation, hypotension and injection site pain significantly decreased in the Remimazolam group (12% vs. 30%, *P* = 0.027; 26% vs. 48%, *P* = 0.023; 4% vs. 48%, *P* < 0.001, respectively). However, there was no statistically significant difference in the incidence of complications such as bradycardia, tachycardia, hypertension respiratory depression, excessive sedation, nausea and vomiting between the two groups.

**TABLE 2:** Adverse events of children (*n* = 50).

Index	Remimazolam group	Control group	*P* Value
Emergency agitation (n) Hypotension (n) Respiratory depression (n) Hypertension (n) Tachycardia (n) Bradycardia (n) Nausea and vomiting (n) Injection site pain (n) Excessive sedation at 30 min postoperatively(n)	6 (12%) 13 (26%) 1 (2%) 16 (32%) 38 (76%) 0 4 (8%) 2 (4%) 0	15 (30%) 24 (48%) 1 (2%) 14 (28%) 34 (68%) 0 3 (6%) 24 (48%) 0	0.027* 0.023* 0.999 0.663 0.610 — 0.999 <0.001* —

Data are expressed as numbers (%).

^*^ *P* < 0.05.

## 4 Discussion

This study found that remimazolam shortened extubation and awakening times and reduced the incidence of emergence agitation, hypotension and injection site pain in preschool children undergoing adenotonsillectomy compared to propofol. However, it was associated with a longer onset time.

Remimazolam is a new ultra-short-acting anesthetics. It produces a sedative effect, mainly by activating the gamma-aminobutyric acid receptors in the ascending reticular activating system, enhancing the inhibition and blocking of cortical and limbic system arousal (Vellinga et al., 2024). This study found that remimazolam shorten the extubation time compared with propofol (12.9 ± 2.2 vs14.5 ± 3.2 min, *P* = 0.005), due to the weak respiratory depression properties of remimazolam. Hu et al. (2022) found that the incidence of respiratory depression with remimazolam was significantly lower in elderly patients undergoing gastroscopy compared with propofol (9.8% vs 17.9%, *P* = 0.042). Remimazolam’s mild respiratory depressant effects facilitate quicker recovery of spontaneous breathing, thereby shortening extubation time in children. However, there is limited information regarding the comparative studies of remimazolam and propofol in children.

We found that the awakening time was shorter in the remimazolam group than that in the Propofol group (19.9 ± 4.7 vs. 21.8 ± 4.5 min, *P* = 0.039). Unlike propofol, remimazolam undergoes organ-independent metabolism to form an inactive metabolite. Remimazolam follows first-order pharmacokinetics, meaning that higher doses or prolonged infusions are unlikely to result in accumulation or extended effects. Moreover, remimazolam in children is characterized by high clearance and a short context-sensitive half-time. In contrast, the metabolism of propofol relies on liver or kidney function in preschool children, the awakening time of propofol was longer compare with remimazolam due to decreased cytochrome P450 enzyme activity in children (Liu et al., 2021). Remimazolam is hydrolyzed by non-specific cholinesterase in plasma without accumulation when continuously infusing remimazolam, and duration of remimazolam was relatively constant. However, Zhou et al. (2022) reported that the awakening time of remimazolam was longer than propofol in adults undergoing bronchoscopy (median 11.00 vs 7.00 min, *P* < 0.001). A systematic review revealed that remimazolam was associated with prolonged extubation times and longer PACU stays without the use of antagonists (such as flumazenil) compared to propofol (Song et al., 2025). This finding contradicts our results, likely due to the decreased cytochrome P450 enzyme activity in preschool children.

The literature indicated that the simulated context-sensitive half-time following a 4-h infusion was 6.8 ± 2.4 min in healthy male volunteers who received remimazolam via continuous intravenous infusion. Loss of consciousness was observed 5 ± 1 min after the infusion began (Schüttler et al., 2020). In the present study, the onset time of remimazolam was 64.3 ± 8.1 s, attributed to different administration modes.

Remimazolam exerts a relatively weak suppressive effect on the cardiovascular system. Propofol is currently the most commonly used intravenous anesthetic in clinical practice. However, it is associated with circulatory depression and hypotension (Qiu et al., 2022; Lan et al., 2024). Compared to propofol, the incidence of hypotension of remimazolam was significantly lower during the induction of general anesthesia, resulting in more stable hemodynamics. Remimazolam demonstrated superiority in terms of hemodynamic stability, and could be rapidly reversed by flumazenil (Zhu X. et al., 2021; Tang et al., 2023). These findings were aligned with our results.

The present study indicated that remimazolam significantly reduced the incidence of emergence agitation, hypotension, and injection site pain. Emergence agitation is a common clinical phenomenon in pediatrics. The pathophysiology of emergence agitation is still unknown. It is related to many factors including, such as preoperative anxiety, unfamiliar environment, use of inhalational anesthetics, and postoperative pain (Kain et al., 2004; Zhu et al., 2021c; Mohkamkar et al., 2014). Cai et al. (2024) found that both a continuous infusion and a single bolus of remimazolam reduced the incidence of emergence delirium in children undergoing laparoscopic surgery. Furthermore, Lu et al. (2025) reported that postoperative intravenous infusion of remimazolam not only reduced the incidence of emergence agitation but also maintained stable hemodynamics in adult patients undergoing nasal surgery. Additionally, Fang et al. (2025) observed that remimazolam was associated with hemodynamic stability and a low incidence of adverse effects in children undergoing elective surgery. These results were well consistent with our findings.

## 5 Conclusion

This study demonstrated that remimazolam not only shortened extubation and awakening times but also reduced the incidence of emergence agitation, hypotension, and injection site pain in preschool children undergoing adenotonsillectomy compared with propofol.

## Data Availability

The raw data supporting the conclusions of this article will be made available by the authors, without undue reservation.
